# Serum Brevican as a Biomarker of Cerebrovascular Disease in an Elderly Cognitively Impaired Cohort

**DOI:** 10.3390/biom14010075

**Published:** 2024-01-07

**Authors:** Rachel S. L. Chia, Karolina Minta, Liu-Yun Wu, Kaung H. T. Salai, Yuek Ling Chai, Saima Hilal, Narayanaswamy Venketasubramanian, Christopher P. Chen, Joyce R. Chong, Mitchell K. P. Lai

**Affiliations:** 1 Department of Pharmacology, Yong Loo Lin School of Medicine, National University of Singapore, Singapore 117600, Singapore; 2 Memory Aging and Cognition Centre, National University Health System, Singapore 117600, Singapore; 3 Future Health Technologies, Singapore–ETH Centre, Campus for Research Excellence and Technological Enterprise (CREATE), Singapore 138602, Singapore; 4 Saw Swee Hock School of Public Health, National University of Singapore, Singapore 117597, Singapore; 5 Departments of Epidemiology and Radiology & Nuclear Medicine, Erasmus University Medical Center, 3015 GD Rotterdam, The Netherlands; 6 Raffles Neuroscience Centre, Raffles Hospital, Singapore 188770, Singapore

**Keywords:** Alzheimer’s disease, brevican, biomarker, cerebrovascular disease, cognitive impairment, vascular dementia

## Abstract

In the brain, the extracellular matrix (ECM) composition shapes the neuronal microenvironment and can undergo substantial changes with cerebral pathology. Brevican is integral to the formation of the ECM’s neuroprotective perineuronal nets (PNNs). Decreased brevican levels were reported in vascular dementia (VaD) but not in Alzheimer’s disease (AD). However, the status of brevican in clinical cohorts with high concomitance of AD pathological burden and cerebrovascular disease (CeVD) is unclear. In this study, 32 non-cognitively impaired (NCI), 97 cognitively impaired no dementia (CIND), 46 AD, and 23 VaD participants recruited from memory clinics based in Singapore underwent neuropsychological and neuroimaging assessments, together with measurements of serum brevican. Association analyses were performed between serum brevican and neuroimaging measures of CeVDs, including white matter hyperintensities (WMHs), lacunes, cortical infarcts, and cerebral microbleeds. Using an aggregated score for CeVD burden, only CIND participants showed lower brevican levels with higher CeVD compared to those with lower CeVD burden (*p* = 0.006). Among the CeVD subtypes assessed, only elevated WMH burden was associated with lower brevican levels (OR = 2.7; 95% CI = 1.3–5.5). Our findings suggest that brevican deficits may play a role in early cerebrovascular damage in participants at risk of developing dementia.

## 1. Introduction

The top two most common causes of dementia in the elderly are Alzheimer’s disease (AD) and vascular dementia (VaD). AD is a progressive neurodegenerative condition characterised by cortical deposition of beta-amyloid (Aβ) plaques and neurofibrillary tangles (NFTs). In contrast, VaD is associated with cerebrovascular diseases (CeVD) and falls under the spectrum of vascular cognitive impairment (VCI). Interestingly, magnetic resonance imaging (MRI) markers of CeVD, including white matter lesions, lacunes, cortical infarcts, and cerebral microbleeds, are also frequently observed in AD brains [1,2,3]. Indeed, the processes of AD and CeVD-associated pathologies are often found together as mixed dementia and share comorbidities and overlapping pathophysiological mechanisms including neurodegeneration, synaptopathology, neuroinflammation, and extracellular matrix (ECM) alterations [4,5,6,7,8,9,10]. Accumulating evidence suggests that these pathophysiological processes may interact in an additive or synergistic manner to exacerbate cognitive impairments [6,11,12]. Interestingly, concomitant AD and CeVD prevalence may be higher in populations from certain geographical regions, such as Asia [6,13], and constitutes an unmet need for clinically useful biomarkers and therapeutic targets. In this context, blood-based markers have the potential to be highly sensitive, specific, and convenient measurements for detecting and monitoring AD and CeVD pathologies [14,15,16]. 

Brevican is a central nervous system (CNS)-specific chondroitin sulphate proteoglycan (CSPG) primarily expressed in astrocytes and neurons [17], and is an integral component of highly organised ECM structures called perineuronal nets (PNNs) [18]. The lattice-like PNNs enwrap CNS parvalbumin-positive (PV+) neurons and regulate the balance between structural stabilisation and synaptic remodelling, which is in turn crucial for maintaining neuronal and synaptic functions [19,20,21]. Any ECM dysfunction may alter ECM remodelling and disrupt the brain’s structural integrity [22]. Since brevican is highly involved in PNNs, it is hypothesised that a brevican deficit may contribute to ECM dysfunction, leading to the progression of brain pathologies [23,24]. 

Recent clinical studies showed decreased brevican in the cerebrospinal fluid (CSF) of VaD, but not AD [25,26,27,28], highlighting its potential associations with CeVD. However, the status of peripheral brevican in AD patients with and without concomitant CeVD is currently unclear. In this study, we measured serum brevican in a Singaporean memory clinic cohort and studied its associations with neuroimaging measurements of amyloid pathology and CeVD [6,13]. 

## 2. Materials and Methods

### 2.1. Study Population

A case–control study design was adopted, with cross-sectional analyses performed on the data. A total of 217 participants from the National University Hospital memory clinic and community in Singapore were recruited as part of the amyloid brain and retinal imaging (ABRI) study cohort (see Supplementary Data S1 for details). Demographic data, imaging, and serum biomarker measurements are presented in Table 1. Informed consent was obtained from patients or their caregivers before study recruitment. Participants underwent detailed medical histories, physical, clinical, and neuropsychological assessments, and neuroimaging. Among the 217 participants, 198 had serum available for brevican measurements and were included in this study. Clinical diagnoses of cognitive impairment, AD, and VaD were based on standardised tests, including a comprehensive neuropsychological test battery, as well as consensus criteria, as previously described [29]. 

### 2.2. Demographic and Risk Factor Assessments 

A detailed questionnaire was administered to collect relevant demographic (age, gender, race, and years of education) and medical information for all participants. Data collected for medical information included risk factors such as hypertension, hyperlipidaemia, diabetes mellitus, and cardiovascular diseases and were classified as present or absent. Hypertension was defined as systolic blood pressure of 140 mm Hg or more and/or diastolic blood pressure of 90 mm Hg or more or a history of antihypertensive medication use. Diabetes mellitus was defined as glycated haemoglobin (HbA1c) of 6.5% or more or being on diabetic medication. Hyperlipidaemia was defined as total cholesterol levels of 4.14 mM or more or being on lipid-lowering medication. Cardiovascular disease was classified as positive medical history of atrial fibrillation, congestive heart failure, and myocardial infarction. Genotyping for apolipoprotein E (*APOE*) ε4 carrier status (presence of at least one *APOE* ε4 allele) was performed as previously described [7].

### 2.3. Neuroimaging

#### 2.3.1. Amyloid PET-MRI Acquisition and Quantification

Amyloid PET imaging was performed at the Clinical Imaging Research Centre of the National University of Singapore using either the [^11^C]Pittsburgh Compound B (PiB) or [^18^F]Flutafuranol amyloid tracer radioligands. A total of 217 subjects underwent a 30 min brain PET scan on an mMR synchronous PET/MR scanner 40 min after intravenous injection of 370 (+/−15%) MBq of [^11^C]PiB or a 20 min brain PET scan on an mCT PET-CT scanner (Siemens Healthineers GmbH, Erlanger, Germany) 50 min after intravenous injection of 185MBq of [^18^F]Flutafuranol (range 166–203 MBq). All images were reconstructed using ordinary Poissonordered subsets expectation maximisation with all corrections applied. Amyloid PET images were independently visually interpreted by three raters blinded to the clinical diagnosis of each subject and following the criteria described previously [30,31]. Using individual parcellated MRI as reference and target region definition based on an in-house developed automated pipeline [32], a global standardised uptake value ratio (SUVr) was derived from the [^11^C]PiB scans. On top of that, cortical Aβ status as binary criteria was derived by merging the equivocal scans with the positive Aβ scans [31].

#### 2.3.2. Brain Atrophy and CeVD MRI Markers

MRI scans were performed on a 3T Siemens Magnetom Trio Tim scanner using a 32–channel head coil at CIRC, NUS. As described previously, the sequences included T1–weighted, fluid-attenuated inversion recovery (FLAIR), and T2–weighted imaging sequences [33] (see Supplementary Data S2 for details). The image preprocessing and tissue classification algorithms used have been previously described [34]. The detection of white matter hyperintensity (WMH, as an indicator for white matter lesions) was achieved using an adapted threshold technique based on the tissue segmentation method where a k-nearest-neighbour classifier was used to classify voxels into CSF, grey matter, and normal-appearing white matter, and subsequently, WMH volume was calculated from these measurements [35]. Brain atrophy was indicated by two parameters: (a) hippocampal volumes which were calculated for the right and left hemispheres, with average volumes measured in millilitres and (b) global cortical thickness which was measured as the shortest distance between the grey/white matter boundary and pial surface at each vertex.

Lacunes were defined as 3–15 mm in diameter lesions, with a low signal on the T1–weighted image and FLAIR, a high signal on the T2–weighted image, and a hyperintense rim with a centre following CSF intensity on FLAIR. Cortical infarcts were defined as focal lesions involving cortical grey matter, a signal following CSF intensity, a hyperintense rim on FLAIR images, and tissue loss of variable magnitude, with prominent adjacent sulci and ipsilateral ventricular enlargement [7]. Using the Brain Observer Microbleed Scale to grade cerebral microbleeds, CMBs were defined as focal, rounded areas of hypointensity (2–10 mm in diameter), with blooming on SWI. CMBs found in the lobar and deep regions were recorded and used as the total number of CMBs [32,36]. 

MRI markers of CeVD were transformed into binary variables and recorded as the presence/absence of ≥2 lacunes, ≥1 cortical infarct, and ≥2 CMBs. The presence of elevated WMH volume was defined at the cut-off of the 50th percentile (median) of WMH volume. “Higher CeVD burden” (CeVD+) was defined as the presence of cortical infarct and/or presence of ≥2 lacunes and/or presence of ≥2 CMBs and/or higher than the 50th percentile of WMH volume. If a participant did not have ≥2 lacunes, ≥2 CMBs or ≥1 cortical infarct and below median WMH volume, they were considered to have “Lower CeVD burden” (CeVD−). 

### 2.4. Serum Brevican Measurements

Non-fasting blood was drawn from study participants into serum separator tubes (SST) and processed by centrifugation at 2000× *g* for 10 min at 4 °C, then stored at −80 °C until use. All samples were subject to only one freeze–thaw cycle. The concentration of serum brevican was measured in duplicate by evaluators blinded to clinical information at the National University of Singapore (NUS). Serum brevican concentrations were measured using the brevican ELISA assay kit (Raybiotech Life, Inc., Peachtree Corners, GA, USA). The minimum detectable concentration of serum brevican is 0.041 ng/mL, with intra- and inter-assay CV% of <10% and <12%, respectively. 

### 2.5. Statistical Analyses

Statistical analyses were performed with IBM SPSS version 26 (IBM Co., Armonk, NY, USA). Group comparisons of continuous demographic variables were performed using one-way analysis of variance (ANOVA) with Bonferroni or Fisher’s least significant difference (LSD) post hoc tests for normally distributed data, and the Kruskal–Wallis test with Dunn’s procedure for skewed distribution data. Chi-square tests were used for categorical variables. Correlation analyses were performed using Spearman’s rank correlation. Serum brevican levels were multiplied by ten before being logarithmically transformed due to the skewed distribution for further analyses. To assess the association between each dichotomous neuroimaging variable (WMH volume, lacune counts, cortical infarct counts, cerebral microbleed counts, and amyloid PET reading) with serum brevican, binary logistic regressions with odds ratios (ORs) and 95% confidence intervals (CI)s were computed. The regression models were performed independently for each neuroimaging variable. Significance was set at *p* < 0.05.

## 3. Results

### 3.1. Participant Characteristics

Among the 198 study participants, 32 (16.2%) individuals were NCI controls, 97 (49.0%) were CIND, 46 (23.2%) were AD, and 23 (11.6%) were VaD patients. Demographic data, neuroimaging, and serum brevican measurements of the study participants are reported in Table 1. Patients with AD and VaD had lower education levels (*p* < 0.001) compared to those with NCI. Patients with AD had higher *APOE* ε4 carrier frequency (*p* = 0.01) and positive Aβ PET read (*p* < 0.001) compared to those with NCI. The prevalence of CeVD was also significantly higher in patients with CIND and VaD (*p* < 0.001) compared to those with NCI. AD patients showed the highest PiB-PET SUVR values (*p* < 0.001) among the diagnostic groups. No significant difference was observed in the prevalence of vascular risk factors (hypertension, diabetes, hyperlipidemia, and cardiovascular diseases) and in serum brevican levels among the groups (Table 1). Serum brevican was not correlated with age (rho = −0.010, *p* = 0.885), years of education (rho = 0.008, *p* = 0.914), WMH volume (rho = −0.106, *p* = 0.138), hippocampal volume (rho = −0.024, *p* = 0.756), global cortical thickness (rho = −0.092, *p* = 0.236), and PiB-PET SUVR (rho = 0.130, *p* = 0.094).

### 3.2. Serum Brevican Concentrations in a Clinical Cohort Stratified by Aβ and CeVD Burden

**Figure 1:**
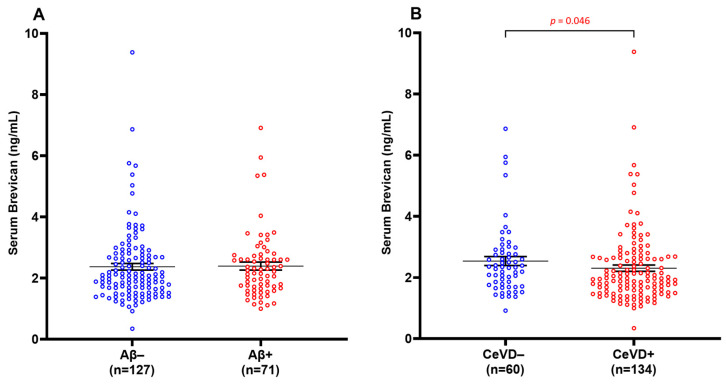
Serum brevican concentrations in a cohort of cognitively impaired participants and those with dementia stratified by (**A**) Aβ positivity and (**B**) CeVD burden. Graph shows mean ± SEM concentration values in ng/mL, with coloured dots indicating individual measurements. CeVD data were missing for four participants. Abbreviations: Aβ, beta-amyloid; CeVD, cerebrovascular disease; SEM, standard error of the mean.

Groups were dichotomised into β-amyloid negative (Aβ−) and β-amyloid positive (Aβ+) groups, and similarly for CeVD− vs. CeVD+. No significant difference in serum brevican levels was observed between the Aβ− and Aβ+ groups (Figure 1A) (*p* > 0.05). Serum brevican was also not related to *APOE* ε4 status (*p* > 0.05). However, there was a significant reduction in serum brevican levels in CeVD+ compared to CeVD− participants (Figure 1B) (*p* = 0.046). 

### 3.3. Serum Brevican Concentrations in a Clinical Cohort Stratified by Clinical Diagnosis and CeVD

**Figure 2:**
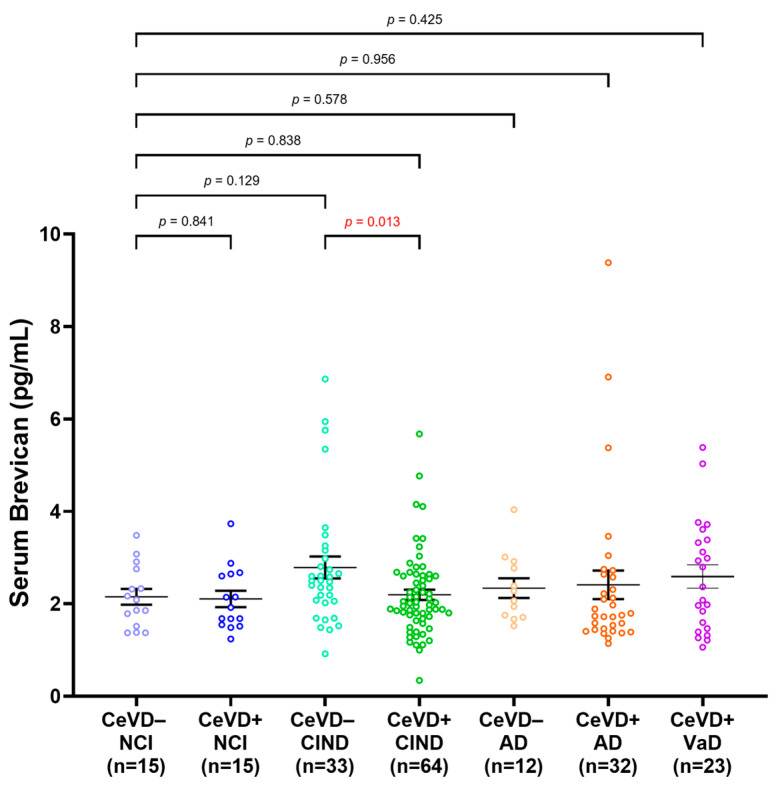
Serum brevican concentrations in a cohort of cognitively impaired participants and those with dementia stratified by clinical diagnoses and CeVD status. Graph shows mean ± SEM concentration values in ng/mL, with coloured dots indicating individual measurements. CeVD data were missing for 2 NCI and 2 AD participants. The group differences were assessed with univariate general linear model using log-transformed serum brevican levels, adjusted for age and sex, with *p*-values representing ANOVA post hoc LSD tests for pairwise group comparisons. Abbreviations: AD, Alzheimer’s disease; Aβ, beta-amyloid; CeVD, cerebrovascular disease; CIND, cognitive impairment, no dementia; NCI, no cognitive impairment; SEM, standard error of the mean.

Serum brevican was next compared among participants stratified by clinical diagnoses and CeVD burden (Figure 2). No significant difference was observed in serum brevican among the groups compared to NCI CeVD−. Serum brevican was significantly lower in the CIND CeVD+ subgroup compared to the CIND CeVD− group (*p* = 0.013), whilst NCI ± CeVD and AD ± CeVD subgroups showed similar, albeit non-significant, trends towards lower brevican in the presence of higher CeVD. The significance was retained within the CIND ± CeVD subgroups after adjustments for age and gender (*p* < 0.05).

### 3.4. Decreased Serum Brevican Is Associated Specifically with Elevated White Matter Hyperintensities 

**Figure 3:**
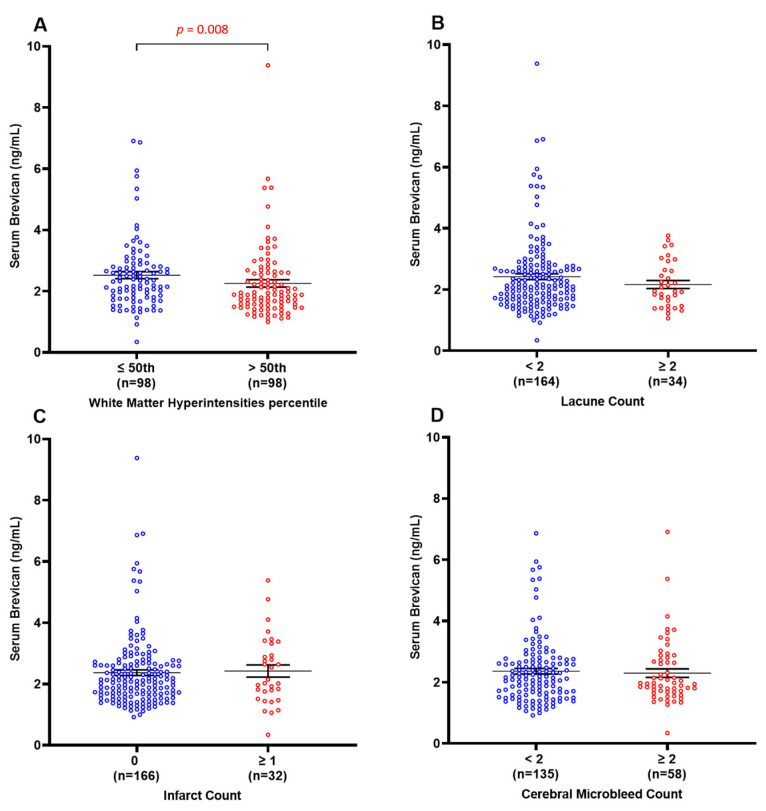
Serum brevican concentrations in a cohort of cognitively impaired participants and those with dementia stratified by the presence of specific CeVD markers. Graph shows mean ± SEM concentration values in ng/mL, with coloured dots indicating individual measurements, stratified by (**A**) WMH volume, (**B**) lacune count, (**C**) infarct count, and (**D**) cerebral microbleeds count. WMH data were missing for two participants. CMBs data were missing for 4 participants. Abbreviations: CeVD, cerebrovascular disease; CMBS, cerebral microbleeds; SEM, standard error of the mean; WMHs, white matter hyperintensities.

Further analyses explored potential associations between the measured serum brevican levels and various MRI markers of CeVD, including WMH, lacunes, infarcts, and CMBs. Figure 3 shows that serum brevican concentrations were significantly lower in subjects with elevated WMH volume (>50th percentile) (*p* = 0.008) but not for the other three neuroimaging features (lacune, infarct, and cerebral microbleed count) (*p* > 0.05 for all). Binary logistic regression models in Table 2 similarly showed significant associations between lower levels of serum brevican (*p* = 0.005; OR = 2.8; 95% CI = 1.4–5.8) and elevated WMH volume in the entire clinical cohort. After adjusting for age, gender, vascular risk factors (i.e., hypertension, diabetes, hyperlipidaemia, and cardiovascular diseases), and the other three neuroimaging features (lacune, infarct, and cerebral microbleed count), a significant association with elevated WMH volume remained for lower serum brevican (*p* = 0.005; OR = 3.0; 95% CI = 1.4–6.4). Serum brevican was not associated with lacune, infarct, and cerebral microbleed counts (*p* > 0.05 for all).

### 3.5. ROC Analyses of Serum Brevican as a Possible Biomarker of Early Vascular Damage

Using receiver operating characteristic (ROC) curve analyses, the utility of serum brevican in distinguishing between CeVD burden in the extant cohort and within CIND participants was assessed. The unadjusted ROC analyses showed an area under the ROC curve (AUC) of 0.590 (sensitivity = 54%; specificity = 67%; 95% CI = 0.5–0.7) for distinguishing CeVD+ from CeVD− individuals in the whole cohort at a cut-off 2.05 ng/mL for serum brevican levels. In contrast, within the CIND subgroup, unadjusted ROC analyses showed an AUC of 0.646 (sensitivity = 55%; specificity = 76%; 95% CI = 0.5–0.8) in distinguishing between CIND CeVD+ and CIND CeVD− groups at a cut-off 2.05 ng/mL for serum brevican levels. These results suggest that serum brevican only has moderate utility in identifying CIND individuals with CeVD.

## 4. Discussion

Using data from a Singaporean elderly cohort who underwent comprehensive neuropsychological and neuroimaging assessments, we explored potential associations of brain amyloid and CeVD with serum brevican. Our main findings are as follows: (1) decreased serum brevican levels were found in CeVD+ compared to CeVD− participants; (2) subgroup analyses showed that the significantly lower serum brevican levels in CeVD+ compared to CeVD− was specifically found in CIND participants; (3) decreased serum brevican levels were specifically associated with white matter hyperintensities. Our study builds upon previous research showing decreased CSF brevican in VaD patients compared to those with NCI [26,28] and extends these findings by reporting a significant association between lower levels of peripheral circulating brevican and elevated WMH volume. However, unlike previous CSF-based studies, we did not detect significant serum brevican alterations in VaD. Possible reasons for the discrepancy include cohort, sampling, and measurement differences. It is also possible that in contrast to CSF, serum brevican is more sensitive to early cerebrovascular changes prior to the sequela of VaD. 

In agreement with previous studies reporting no brevican alteration in AD serum or the brain [28,37], the current study did not detect significant differences in serum brevican levels in groups stratified by amyloid status. Taken together with the above-described findings in CeVD subgroups, our results suggest that lower serum brevican levels may reflect vascular pathologies independent of AD pathology. Although the exact mechanisms underlying serum brevican decreases have not been fully elucidated, they likely reflect the upregulation of vascular remodelling. Indeed, the occurrence of CeVD in predementia stages (i.e., CIND) may lead to increased proteolytic activity, resulting in the cleavage and subsequent release of brevican fragments into the bloodstream. For example, brevican is a known substrate of ECM proteases such as a disintegrin and metalloproteinase with thrombospondin motifs (ADAMTS) as well as matrix metalloproteinases (MMPs) [38], which in turn are known to be dysregulated in the presence of vascular pathology [5,39,40,41]. Therefore, proteolytic cleavage and subsequent degradation of ECM proteins including brevican may reduce the integrity of the brain ECM which supports structural plasticity. Furthermore, the dysregulation of ECM proteases may contribute to other CeVD-related pathophysiological processes including neuroinflammation and endothelial or perycytic damage, thereby exacerbating CeVD-initiated synaptic dysfunction and neurodegeneration, resulting in dementia [42,43]. In line with this postulate, brevican-deficient mouse models showed impaired forms of hippocampal long-term potentiation [44], altered function of PV+ interneurons [45], and impaired hippocampus-dependent spatial or contextual acquisition learning [46], while senescence-accelerated mouse strains showed reduced brevican-positive PNNs [47]. Taken together, these findings indicate the possible involvement of brevican in cognitive functions. 

Interestingly, amongst the CeVDs assessed (WMH, lacunes, cortical infarcts, and CMBs), serum brevican was negatively associated only with WMH, which is a neuroimaging marker of white matter lesions. As white matter lesions represent early small vessel disease preceding lacunes and infarcts, but which is nevertheless associated with long-term risks of vascular cognitive impairment and dementia [48], our findings support the involvement of brevican alterations in early stages of cerebrovascular pathology, as well as the postulate of brevican replacement as a potential therapeutic strategy. However, further studies are required to understand the mechanisms underlying brevican’s role in cerebrovascular health and disease. 

The strengths of the current study include the following: (1) the use of comprehensive neuropsychological assessments to diagnose cognitive impairment and dementia properly; (2) the use of well-established neuroimaging measurements for amyloid and CeVD pathology; and (3) the inclusion of covariates such as age, gender, education, and *APOE* ε4 carrier. However, our study also has several limitations. Firstly, the study size was relatively small, leading to small subgroup numbers, especially when the clinical cohort was stratified by clinical diagnosis and CeVD, leading to non-significant trends in some of the observations. Furthermore, the cross-sectional design of the study did not allow for the examination of temporal associations between serum brevican and the progression of WMH burden or cognitive impairment, giving rise to the need for follow-up longitudinal studies. Also, potential confounding effects of other ECM proteins, such as MMPs, ADAMTS, and tissue inhibitors of metalloproteinases (TIMPs), were not addressed in this study. Since TIMPs are known to be inhibitors of MMPs and ADAMTS and are associated with VaD [49,50], it would be of interest to assess the potential roles of TIMPs and other ECM components in cerebrovascular disease pathogenesis. As this study focuses on cerebral vascular disease and the importance of brevican on the cerebral extracellular matrix, future studies investigating MRI assessments of intracerebral haemorrhages, dilated perivascular spaces, or BBB leakage and their association with serum brevican would strengthen the observations made in this study. In this study, the median cut-off for WMH volume was performed to separate participants into ‘elevated’ and ‘low’ WMH loads, as there is no current consensus on established cut-offs for WMH volume [8]. Although this method has been used in multiple publications [7,8], the exact cut-off values may differ across cohorts. Finally, while this study shows that serum brevican is a possible biomarker for early CeVD pathology in predementia groups, the moderate ROC values obtained suggest a need for additional studies to evaluate ECM or other biomarkers that can complement brevican in a multi-marker panel in improving sensitivity and specificity.

## 5. Conclusions

Our findings suggest that decreased serum brevican may be associated with early cerebrovascular damage (i.e., white matter lesions) in an elderly predementia (CIND) group. Further assessments would be required to assess the role of brevican in cerebrovascular health and disease, as well as the clinical utility of serum brevican in identifying predementia patients with CeVD. 

## Figures and Tables

**Table 1:** Baseline demographics and disease factors of study participants.

	NCI	CIND	AD	VaD	*p*-Value
*Demographics*					
Maximum n	32	97	46	23	
Age, y, mean (SD)	76 (4)	76 (6)	77 (8)	75 (9)	0.767
Female, n (%)	21 (66)	49 (51)	36 (78) ^g^	8 (35) ^h^	**0.001**
Education, y, mean (SD)	11 (5)	8 (5)	5 (5) ^a,b^	4 (4) ^a^	**<0.001**
APOE ε4 carrier, n (%)	3 (9)	25 (26)	20 (44) ^f^	7 (30)	**0.01**
Hypertension, n (%)	23 (72)	73 (75)	35 (76)	21 (91)	0.349
Diabetes, n (%)	5 (16)	32 (33)	10 (22)	9 (39)	0.126
Hyperlipidaemia, n (%)	26 (81)	70 (72)	29 (63)	19 (83)	0.213
Cardiovascular diseases, n (%)	1 (3)	10 (11)	1 (2)	3 (14)	0.162
*Neuroimaging*					
Presence of ≥2 lacunes, n (%)	2 (6)	16 (17)	3 (7) ^g^	13 (57) ^f,g,h^	**<0.001**
Presence of cortical infarct, n (%)	2 (6)	15 (16)	6 (13)	9 (39) ^f^	**0.009**
Presence of ≥2 CMBs, n (%)	10 (33)	24 (25)	17 (40)	7 (30)	0.348
Higher 50th WMH, n (%)	8 (25)	49 (51)	26 (58) ^f^	15 (68) ^f^	**0.007**
WMH volume, median (IQR), mL	1.4 (3.9)	3.7 (11.0) ^c^	5.3 (10.7) ^c^	14.6 (21.6) ^c^	**0.001**
Hippocampal volume, median (IQR), mL	7.1 (0.9)	6.3 (1.8)	5.0 (1.0) ^c,d^	6.0 (1.1) ^c,e^	**<0.001**
Global cortical thickness, median (IQR), mm	2.4 (0.2)	2.3 (0.1)	2.2 (0.2) ^c,d^	2.3 (0.2)	**<0.001**
Positive Aβ PET read, n (%)	4 (13)	32 (33)	31 (67) ^f,g^	4 (17) ^h^	**<0.001**
PiB-PET SUVR, median (IQR)	1.1 (0.1)	1.2 (0.4)	1.9 (0.7)	1.2 (0.3)	**<0.001**
Elevated CeVD, n (%)	15 (50)	64 (66)	32 (73)	23 (100) ^f,g^	**0.001**
Serum brevican, median (IQR), ng/mL	2.0 (1.1)	2.2 (0.9)	2.0 (1.2)	2.4 (1.9)	0.476

**Bold, red font** denotes significant *p*-values from Chi-square tests (gender, APOE status, and neuroimaging markers for white matter hyperintensities, lacunes, and infarct) for categorical variables and from one-way ANOVA (age and education) or Kruskal–Wallis tests (WMH volume, PiB-PET SUVR, and serum brevican) for normally distributed or skewed continuous variables, respectively. White matter hyperintensity data were missing for 1 AD and 1 VaD participant. Diabetes data were missing for 1 AD participant. Cardiovascular disease data were missing for 3 CIND and 1 VaD participants. CeVD data were missing for 1 AD participant. CMBs data were missing for 2 NCI and 3 AD participants. Hippocampal volume and cortical thickness data were missing for 16 NCI, 1 CIND, 11 AD, and 3 VaD participants. PiB-PET SUVR data were missing for 16 NCI, 11 AD, and 4 VaD participants. Abbreviations: Aβ, amyloid beta; AD, Alzheimer’s disease; CeVD, cerebrovascular disease; CIND, cognitive impairment, no dementia; CMBs, cerebral microbleeds; NCI, no cognitive impairment; SD, standard deviation; SUVR, standardised uptake value ratio; VaD, vascular dementia. ^a^ Significantly different from NCI (one-way ANOVA with post hoc Bonferroni tests *p* < 0.05). ^b^ Significantly different from CIND (one-way ANOVA with post hoc Bonferroni tests *p* < 0.05). ^c^ Significantly different from NCI (Kruskal–Wallis with post hoc Dunn’s test *p* < 0.05). ^d^ Significantly different from CIND (Kruskal–Wallis with post hoc Dunn’s test *p* < 0.05). ^e^ Significantly different from AD (Kruskal–Wallis with post hoc Dunn’s test *p* < 0.05). ^f^ Significantly different from NCI (Chi-square *t*-tests *p* < 0.05). ^g^ Significantly different from CIND (Chi-square *t*-tests *p* < 0.05). ^h^ Significantly different from AD (Chi-square *t*-tests *p* < 0.05).

**Table 2:** Associations of serum brevican with specific CeVD neuroimaging markers.

CeVD Binary Outcome Variables Using Binary Logistic Regression								
Serum Brevican (Tertiles)	WMH (>50th Percentile) (n = 196)		Presence of ≥2 Lacunes (n = 198)		Presence of Cortical Infarcts (n = 198)		Presence of ≥2 CMBs (n = 194)	
	OR (95% CI)	*p*	OR (95% CI)	*p*	OR (95% CI)	*p*	OR (95% CI)	***p***
*Model 1*								
Lowest	**2.8 (1.4–5.8)**	**0.005**	1.3 (0.5–3.3)	0.626	0.5 (0.2–1.3)	0.164	1.0 (0.4–2.1)	0.907
Middle	1.5 (0.8–3.1)	0.235	1.3 (0.5–3.4)	0.550	0.6 (0.2–1.4)	0.207	1.0 (0.5–2.2)	0.926
Highest	1		1		1		1	
*Model 2*								
Lowest	**2.8 (1.4–5.8)**	**0.006**	1.4 (0.5–3.7)	0.522	0.5 (0.2–1.4)	0.192	1.0 (0.4–2.1)	0.958
Middle	1.5 (0.8–3.1)	0.223	1.4 (0.5–3.7)	0.479	0.6 (0.2–1.5)	0.248	1.0 (0.5–2.2)	0.909
Highest	1		1		1		1	
*Model 3*								
Lowest	**3.0 (1.4–6.4)**	**0.005**	1.1 (0.4–3.2)	0.853	0.4 (0.1–1.2)	0.099	0.8 (0.3–1.8)	0.522
Middle	1.6 (0.8–3.5)	0.197	1.4 (0.5–4.1)	0.518	0.3 (0.1–1.0)	0.050	0.9 (0.4–2.1)	0.743
Highest	1		1		1		1	

Associations between serum brevican and specific CeVD neuroimaging markers (WMH, lacune, cortical infarcts, and CMBs) are expressed as OR values with 95% CI. **Bold, red fonts** denote statistically significant associations derived from binary logistic regression at *p* < 0.05. WMH data were missing for 2 participants. CMBs data were missing for 4 participants. *Model 1*: Unadjusted; *Model 2*: Adjusted for age and gender; *Model 3*: Adjusted for age, gender, hypertension, diabetes, hyperlipidaemia, cardiovascular diseases, and the other three MRI markers. Interpretation: when **OR > 1** (*p* < 0.05), each 2-fold (1 unit of Log2 [serum brevican]) decrease in serum brevican level was associated with OR times higher likelihood of obtaining the respective cognitive outcome. Abbreviations: CeVD, cerebrovascular disease; CI, confidence interval; CMBs, cerebral microbleeds; OR, odds ratio; WMHs, white matter hyperintensities.

## Data Availability

Anonymised data derived from this study may be provided by the corresponding author upon reasonable request.

## Supplementary Materials

The following supporting information can be downloaded at: https://www.mdpi.com/article/10.3390/biom14010075/s1, Supplementary Data S1: Flowchart of Study Participants; Supplementary Data S2: Details for MRI protocol.
