# Supplementary material for: Serum Brevican as a Biomarker of Cerebrovascular Disease in an Elderly Cognitively Impaired Cohort
Source: Biomolecules. 2024 Jan 7;14(1):75. doi: 10.3390/biom14010075 (PMC10813026; doi:10.3390/biom14010075)
Supplement: Supplementary file 1 [file biomolecules-14-00075-s001.zip › biomolecules-2774430-supplementary.pdf]

Chia *et al.* Supplementary information

Supplementary Data S1: Flowchart for study participant selection

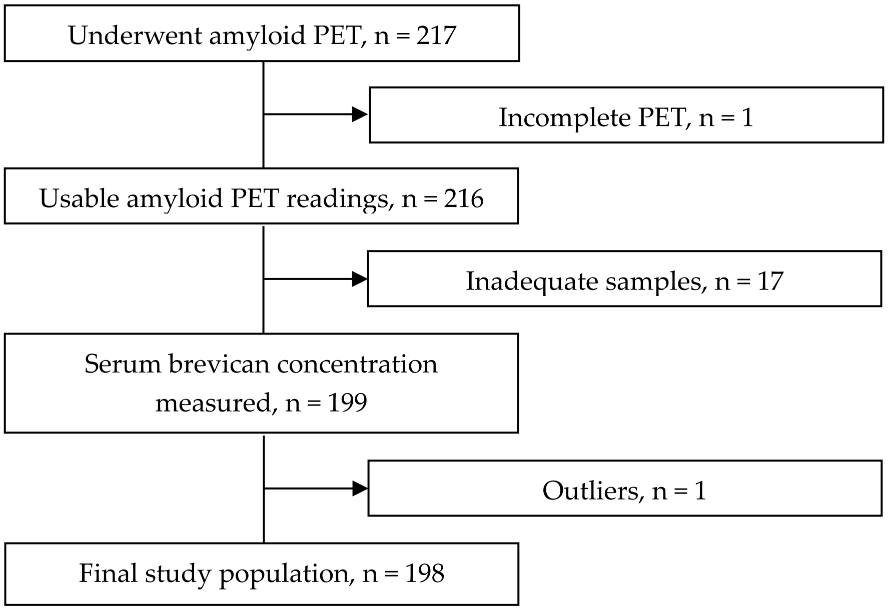

A schematic diagram illustrating the study population used to measure serum brevican. A total of 217 patients underwent an amyloid positron emission tomography (PET) scan between April 2016 and April 2019 at the Memory, Aging and Cognition Center, National University Health System, with 216 patients having usable PET scan readings. Out of the 216 samples with complete PET scan readings, 199 samples were used to measure levels of serum brevican, with 17 inadequate samples.

Supplementary Data S2: Details for MRI protocol

The standardized protocol included a three-dimensional (3D) T1-weighted (1.0 X 1.0 X 1.0 mm3 voxels; repetition time (TR) 2300 ms; echo time (TE) 1.9 ms; inversion time(TI) 900 ms; flip angle 9°; matrix 256 X 256), a 2D multislice T2-weighted (1.0 X 1.0 X 3.0 mm^3 voxels; TR 3000 ms; TE 10.1 ms; matrix 247 X 256), a 2D multislice FLAIR (1.0 X 1.0 X 3.0 mm3; TR 9000 ms; TE 82 ms; TI 2500 ms; matrix 232 X 256), and a 2D multislice T2*-weighted image (1.0 X 1.0 X 1.5 mm^3 voxels; TR 27 ms; TE 20 ms; flip angle 15°; matrix 192 X 256), for the assessment of markers of small vessel disease (white matter hyperintensities and lacunes) [1].

Reference:

1. van Veluw, S.J.; Hilal, S.; Kuijf, H.J.; Ikram, M.K.; Xin, X.; Yeow, T.B.; Venketasubramanian, N.; Biessels, G.J.; Chen, C. Cortical microinfarcts on 3T MRI: Clinical correlates in memory-clinic patients. *Alzheimers Dement* **2015**, *11*, 1500-1509, doi:10.1016/j.jalz.2014.12.010.
